# P32 Carbapenem-resistant *Acinetobacter* spp as a major pathogen in early-onset neonatal sepsis among hospitalized neonates in southern Pakistan

**DOI:** 10.1093/jacamr/dlaf230.039

**Published:** 2025-12-04

**Authors:** Asima Shahid Sabzwari, Fatima Mir, Sadia Shakoor

**Affiliations:** Department of Pathology & Laboratory Medicine, The Aga Khan University, Karachi, Pakistan; Department of Paediatrics & Child Health, The Aga Khan University, Karachi, Pakistan; Department of Pathology & Laboratory Medicine, The Aga Khan University, Karachi, Pakistan

## Abstract

**Background:**

Sepsis is a major cause of neonatal mortality especially in Low-and-middle-income (LMIC) settings. Pakistan contributes heavily to the global neonatal mortality burden with rates as high as 42 per 1000 live births reported recently. Neonatal sepsis is a major contributor to the high neonatal mortality rate in Pakistan, with a substantial proportion attributable to sepsis. Lack of diagnostic modalities makes pathogen aetiology of bacterial sepsis difficult to determine.

**Objectives:**

To present pathogen proportions among blood culture-positive cases of early-onset (≤3 days) neonatal sepsis in southern Pakistan.

**Methods:**

We analysed archived data from a central, internationally accredited laboratory. The laboratory serves southern Pakistan through over 200 peripheral collection units; BACT-Alert is used for blood culture testing and monitoring and susceptibilities are performed using CLSI standards using broth microdilution (colistin) and Vitek and/ or disc diffusion methods (other antibiotics). Data were retrieved from the laboratory’s Laboratory Information System (LIS) for 2018–2022. Contaminants (including coagulase negative staphylococci CoNS) and duplicates were removed. Laboratory data were analysed for frequency distributions. Confidence intervals (CI) were calculated using Prism v 10. Clinical correlates and demographic/ facility data were not available.

**Results:**

From 2018 to 2022, 45236 neonatal blood cultures from neonates <3 days old were processed at the study laboratory of which 23061(50.97%) were positive. After removal of contaminants (*Bacillus* spp, *Micrococcus* spp, corynebacteria, CoNS and *Streptococcus viridans*), 2834 cultures from 2832 patients were included. *Acinetobacter* spp was the leading pathogen causing bacterial EOS (22% of episodes; 95% CI 20.5–23.6) followed by *Serratia* spp. (12.3% episodes, 95% CI 11.2–13.6). Among Acinetobacter causing EOS, 72% (95% CI 68.4–75.4) were carbapenem resistant (CR). Of CR Acinetobacter spp (CRAs), 78% (95% CI 73.9–81.6) were MDR where colistin remaining as the only treatment option. 0.4% of CRAs were colistin resistance.

**Conclusions:**

*Acinetobacter* spp have emerged as the predominant pathogen among culture-positive EOS in neonates from southern Pakistan, with high rates of CRAs. Occurrence within 72 h of birth reflects early acquisition and poor infection prevention in birthing facilities. High drug resistance necessitates prioritized research into mechanisms of resistance in CRAs causing EOS, clinical phenotyping of affected neonates, and urgent clinical trials of new antibiotics such as sulbactam-durlobactam and rifamycins among neonates.

